# Response to Mass-Casualty Incidents and Outbreaks: A Prehospital Disaster Training Package Developed for the National Emergency Medical Service in Sierra Leone

**DOI:** 10.1017/S1049023X22001029

**Published:** 2022-10

**Authors:** Marta Caviglia, José Alberto da Silva-Moniz, Francesco Venturini, Amara Jambai, Matthew Jusu Vandy, Abdul Rahman Wurie, Moi T. Sartie, Giovanni Putoto, Luca Ragazzoni

**Affiliations:** 1. CRIMEDIM – Center for Research and Training in Disaster Medicine, Humanitarian Aid, and Global Health, Università del Piemonte Orientale, Novara, Italy; 2. Doctors with Africa – CUAMM, Padova, Veneto, Italy; 3. Ministry of Health and Sanitation, Sierra Leone; 4. The National Emergency Medical Service – NEMS, Ministry of Health and Sanitation, Freetown, Sierra Leone; 5. Department of Sustainable Development and Ecological Transition, Università del Piemonte Orientale, Vercelli, Italy

**Keywords:** disaster medicine training, Emergency Medical Service, mass-casualty incidents, outbreaks, Sierra Leone

## Abstract

Sierra Leone is a country highly prone to disasters, still recovering from the catastrophic 2014 Ebola epidemic. In 2018, the country launched its first National Emergency Medical Service (NEMS) aiming to strengthen the provision of essential health services to the population with the long-term goal of creating a resilient health system able to effectively respond to and recover from emergencies. The Center for Research and Training in Disaster Medicine, Humanitarian Aid, and Global Health (CRIMEDIM), together with the Italian NGO Doctors with Africa (CUAMM), under the direct supervision of the Ministry of Health and Sanitation (MoHS), developed a prehospital Disaster Training Package (DTP) to be delivered to all NEMS personnel to boost the prehospital management of mass-casualty incidents (MCIs) and outbreaks. The DTP included a first phase in which NEMS local trainers underwent a training-of-trainers (ToT) course, enabling them to deliver cascade trainings to 16 district ambulance supervisors, 441 paramedics, 441 ambulance drivers, and 36 operators working in the NEMS operation center. This on-going training package represents the first Disaster Medicine training course for prehospital health professionals in Sierra Leone.

## Introduction

In the last three decades, the African continent experienced over 2,000 disasters, a trend that is likely to continue given the rapid and unplanned urban growth and the escalating impact of climate change.^1^ Sierra Leone, one of the least developed low-income countries, ranks among the ten African countries reporting the highest disaster death toll in the past 20 years as a consequence of a number of disastrous events, including the regional epidemic of Ebola Virus Disease in 2014 and a series of mass-casualty incidents (MCIs) resulting from torrential rains, floods, and landslides.^2^ In 2015, the government of Sierra Leone issued a post-Ebola recovery plan with the ultimate goal of building a resilient national system, enabling the health sector to provide essential health services, and to develop an integrated disaster risk management system.^3^ As part of the essential health services package, the plan envisaged to improve the national prehospital referral transport system, a goal that was achieved in 2018 with the official launch of the first National Emergency Medical Service (NEMS).^4,5^ The NEMS is a coordinated prehospital referral system that entails a fleet of 84 ambulances, 441 paramedics, and 441 prehospital care drivers working to provide timely prehospital care and transportation of patients to the nearest referral hospital under the supervision of 16 district ambulance supervisors and with the support of 36 operation center (OC) operators. Before taking service, NEMS personnel underwent a series of ad-hoc basic training courses developed by the Center for Research and Training in Disaster Medicine, Humanitarian Aid, and Global Health (CRIMEDIM; Università del Piemonte Orientale; Novara, Italy) and delivered with the support of the Italian NGO Doctors with Africa (CUAMM; Padova, Italy) under the direct supervision of the Ministry of Health and Sanitation (MoHS; Freetown, Western Area, Sierra Leone).^4^ The courses embraced different topics, including the management of medical, trauma, obstetrics, gynecology, and pediatric emergencies and Basic Life Support and resuscitation maneuvers without the support of automated external defibrillator.^4^ In addition, a series of refresh courses have been delivered to all NEMS personnel to improve their technical and attitudinal performances, with specific focus in those areas where gaps in knowledge, attitude, and practice were highlighted.

In its first three years of service, the NEMS has been challenged by a series of events that tested its resilience, including the 2019 Lassa Fever outbreak and the COVID-19 pandemic, the latter requiring a number of structural adaptation to ensure both the delivery of routine services and the proper management of COVID-19 patients.^6^ A COVID-19 Special Training has also been provided to the prehospital health care teams and to the operators working at the NEMS OC focusing on the correct use of personal protective equipment; infection, prevention, and control procedures; case definition; triage; and dispatch procedures.^6^ In line with the national recovery plan^3^ and according to the governmental willingness to reinforce disaster risk management institutions and capacities in the country,^7^ in 2021, CRIMEDIM and CUAMM with the support of the MoHS developed a training package with the goal of strengthening the capacity of the NEMS to manage MCIs and respond to outbreaks. The aim of this paper is to describe the prehospital Disaster Training Package (DTP) implemented for the NEMS in Sierra Leone.

## Report

### Training Needs and Learning Methodology

The DTP was designed following the six-step approach to curriculum and training development^8^ with the ultimate goal of creating a workforce comprising qualified emergency responders with specific professional competencies to respond to outbreaks and MCIs. Since NEMS represents the first structured prehospital Emergency Medical Service of Sierra Leone, there was no previous experience in disaster and MCI training and education for prehospital providers in the country. In fact, to the best of the authors’ knowledge, no similar training existed in the African continent at the time of writing of this manuscript, a gap that the World Health Organization (Geneva, Switzerland) Emergency Medical Teams initiative has started addressing in 2021 with the first Mass-Casualty Management course held in Ethiopia which, however, focused mainly on hospital response.^9^ Therefore, to set the targeted learning objectives of the DTP, existing Disaster Medicine curricula designed by CRIMEDIM and other existing courses^10,11^ were reviewed and adapted to produce a training package tailored to the local needs and based on local resources and capabilities. As such, the development process had to consider: (1) the country institutional architecture and national mechanism of response to disasters and MCIs; (2) the NEMS organizational structure; (3) the high burden represented by outbreaks and epidemics; (4) the medical resources present on the ambulances and current medical competences of the NEMS paramedics; and (5) the need to train all NEMS prehospital personnel, thus including approximately 1,000 providers, within a one-year timeframe. To guarantee the capillarity of the DTP throughout the 16 districts of the country and to enable its sustainability over time, the presence of the NEMS national trainers was leveraged, a pool of seven qualified trainers with health backgrounds, specifically as registered nurses or community health officers, responsible for all the NEMS educational activities. Therefore, the first task of the DTP was to develop a one-week training-of-trainers (ToT) course to equip national trainers with both basic knowledge in Disaster Medicine and the necessary skills to transfer this knowledge to all NEMS personnel through a peer-assisted learning (PAL) approach.^12^ The ToT courses aimed to prepare national trainers to critically replicate and lead the same types of exercises, promoting learners’ engagement, reflective practice, critical thinking, and skill acquisition. The second task was to develop a one-week cascade training to be delivered by national trainers to district ambulance supervisors, prehospital teams, and OC operators in the 16 districts of the country. The utmost goal of the cascade training activities was to help prehospital teams and OC operators familiarize with disaster concepts, but also to improve their attitudinal, behavioral, and technical performance, strengthening their existing professional skills and developing context-specific capacities to achieve an effective team performance. The educational strategy adopted included a blended methodology based on adult learning principles, combining traditional classroom teaching methodologies with practical exercises, group discussions, table-top simulations, and drills using mannequins and role players. The curriculum has then been presented to the MoHS representatives at NEMS to obtain support and financing and to identify and address potential barriers to its implementation. Lastly, an evaluation tool was designed to assess the individual performance of learners in a summative way, thus evaluating knowledge retention, and to assess their participation and awareness during the practical sessions.

### Curriculum and Training Structure

In Table 1, the ToT course curriculum is reported, including modules, learning objectives, teaching methods, and time allocated. Trainees were also exposed to a drill reproducing a mass-casualty scenario in which they were asked to perform specific tasks regarding scene assessment, proper communication and reporting to health authorities, primary triage using the Simple Triage and Rapid Treatment (START) algorithm,^13^ and evacuation procedures. The objective of the drill was to exercise the newly acquired skills inside a realistic scenario, both as individuals and within the team, under the supervision of expert evaluators in charge of observing trainees’ performances and leading a post-drill debriefing session. The course was delivered by two qualified training managers with backgrounds in disaster and emergency medicine, supported by CRIMEDIM’s experts. The ToT course comprised a final examination that consisted of 24 multiple-choice questions to assess content knowledge, and test results were expressed as a score out of 100 with a minimum passing score of 60. Trainees’ participation and awareness during practical sessions were also evaluated using a one-to-five score. Participation was defined as “active engagement with course content, faculty, and fellow students” while trainees’ awareness encompassed a combination of social awareness (related to social connections within the group), task awareness (related to the steps needed to complete tasks), and concept awareness (related to the trainees existing knowledge in respect to the tasks).^14^ At the end of the course, the national trainers with the support of training managers, CRIMEDIM’s experts, and relevant stakeholders reviewed the cascade trainings to be delivered to NEMS paramedics, ambulance drivers, and OC operators. A group discussion was held to agree on the topics to be included, which comprised the same modules delivered in the ToT course in addition to a review session focusing on NEMS communication and handover procedures. A final examination consisting of 24 multiple-choice questions and a course evaluation questionnaire were also produced.

**Table 1.** Overview of the Training-of-Trainers Curriculum within the Prehospital Disaster Training Package Developed for the National Emergency Medical Service in Sierra Leone

Module	Learning Objectives	Teaching Methods	Time
Introduction to Disaster Medicine	To understand the main features and functional attributes of disasters To explain the common characteristics of disasters and MCIs To understand the impact of disasters and MCIs To get acquainted with concepts related to disaster risk (ie, hazard, exposure, vulnerability, capacity) To understand and describe the different component of the disaster cycle (ie, response, recovery, preparedness, mitigation)	Classroom lecture	1hour
Triage During MCIs	To understand the basic principles of triage in a context of multiple casualties and limited resources To describe the key concepts related to triage during MCIs To get acquainted with the START algorithm To efficiently perform the START algorithm	Classroom lecture; Practical session: table-top exercise, group discussions, live simulations	6hours
Prehospital Response to MCIs	To understand the difference between different MCIs according to type, setting, and events To perform scene assessment and gather the necessary information to deliver the initial report and alert the system of impending MCI To perform scene size-up and identify different areas (ie, advanced command post, patient collecting, and loading areas) To understand basic concepts of communication during MCIs To establish roles within the team, understanding the importance of the chain of command and control To understand the importance of a coordinated scene evacuation	Classroom lecture; Practical session: table-top exercise, group discussions, live simulations	8hours
Infectious Diseases	To explain the difference between outbreaks, epidemics, and pandemics To get acquainted with International Health Regulations To describe the role of infectious diseases as disasters To describe the main features and risks pertaining the occurrence of infectious diseases after a natural or technological disaster	Classroom lecture	1hour
IPC & PPE	To understand and explain basic concepts pertaining to the infectious disease transmission cycle (ie, agent, reservoir, modes of transmission) To understand and describe the standard precautions to protect operators and prevent operators from spreading infections among patients (ie, hand hygiene, use of PPE, respiratory hygiene and cough etiquette, sharps safety, clean and disinfected environmental surfaces) To review and refresh existing COVID-19 knowledge on policy, procedures, and protocols To be familiar with all parts of PPE and know how to don and doff To understand the decontamination process	Classroom lecture Practical session	6hours
National Response Mechanism to MCIs and Outbreaks	To get familiar with national institutional architecture to manage disasters and emergencies in Sierra Leone To understand the different steps pertaining the national response mechanism in case of MCI and outbreaks To understand the role and responsibilities of NEMS within the national response mechanism	Classroom lecture	2hours
Planning of Cascade Trainings	To get acquainted with the practical sessions to be critically led during the cascade training To understand how to perform effective debriefing sessions after simulation activities To discuss the cascade training schedule according to NEMS operational needs To plan the cascade training activities and material	Group work and discussion	12hours

Abbreviations: MCI, mass-casualty incidents; START, Simple Triage and Rapid Treatment; IPC, infection prevention and control; PPE, personal protective equipment; NEMS, National Emergency Medical Service.

## Outcomes

Starting on July 19, 2021, the ToT course was delivered to the seven national trainers. All trainers successfully passed the final examination and achieved high scores in the practical sessions, demonstrating active participation, commitment to the project, and good awareness (Table 2). The use of a hybrid learning approach featuring frontal lectures and practical sessions, a modality that has already been adopted during the delivery of NEMS basic training courses,^4^ allowed to achieve excellent results concerning students’ engagement and knowledge retention. Following the ToT course, the series of cascade trainings started on August 2, 2021, delivered by the just-trained national trainers under the direct supervision of the two training managers. After three-month stop due to financial issues related to delays in external financing, the cascade trainings are currently on-going with the objective of reaching 1,000 NEMS prehospital providers by the end of the year.

**Table 2.** Evaluation of the NEMS Local Trainers Exposed to the Training-of-Trainers Course on Outbreaks and Mass-Casualty Incidents

	Final Examination	Practical Sessions	
		Participation	Awareness
Trainer 1	75	5	5
Trainer 2	87	5	5
Trainer 3	84	5	5
Trainer 4	75	3	4
Trainer 5	71	4	4
Trainer 6	75	4	5
Trainer 7	80	4	4

Note: The final written examination included 24 multiple choice questions with a cut-off score for passing of 60 out of 100. Practical sessions were evaluated with a 1 to 5 score.

Abbreviation: NEMS, National Emergency Medical Service.

## Discussion and Conclusions

The NEMS’ DTP is the very first Disaster Medicine training course delivered to prehospital health care providers in Sierra Leone. The curriculum development process followed a concrete educational framework,^8^ and the curriculum was tailored according to local needs. The adoption of a PAL approach, a modality that has been successfully implemented in several training programs for health professionals and also in the delivery of disaster medicine courses,^15–17^ was beneficial both for NEMS national trainers and trainees. Indeed, the former had the chance to improve their individual competencies and skills, boosting their self-confidence and autonomy in the provision of training activities, while the latter had the possibility to learn in a “social and cognitive congruent” environment, where trainers and trainee sharing the same social role feel more encouraged to express informally and exchange ideas.^18,19^ The abovementioned considerations indicate that the provision of the DTP to all NEMS personnel has the potential to improve Disaster Medicine culture among health professionals in Sierra Leone. While education and training are the cornerstones of disaster preparedness and response, results in the literature clearly point out the lack of Disaster Medicine trainings in medical schools world-wide,^20^ a deficiency that contributes to leaving health professionals unprepared when facing the consequence of disastrous events, which can rapidly overwhelm local resources and the ability to deliver comprehensive medical care. Few sporadic steps were made in the past years to provide Disaster Medicine education in African countries,^9^ and in most cases, involved health professionals in South Africa, the most developed country of the continent.^21,22^

The authors strongly believe that the DTP delivered to NEMS personnel represents an important step towards the strengthening of disaster risk management efforts in the country, with the possibility to be extended to other emergency responders such as the police and fire department, as well as all the partners involved in the national response plan. Moreover, this experience has the potential to expand beyond its national borders and to foster the implementation of similar projects at the global level.
